# Protocol for the Emory University African American maternal stress and infant gut microbiome cohort study

**DOI:** 10.1186/s12887-019-1630-4

**Published:** 2019-07-22

**Authors:** Patricia A. Brennan, Anne L. Dunlop, Alicia K. Smith, Michael Kramer, Jennifer Mulle, Elizabeth J. Corwin

**Affiliations:** Department of Psychology, Emory University, 36 Eagle Row, Atlanta, GA 30322 USA

**Keywords:** Stress, Perinatal, Microbiome, Infant, Neurodevelopment

## Abstract

**Background:**

The microbial population of the human gut (the gut microbiome) is an integral cog in the bidirectional communication axis that exists between the gastrointestinal tract and the central nervous system. African American infants disproportionately experience multiple, overlapping vulnerabilities such as preterm birth and formula rather than breast feeding that may disrupt the development of the infant microbiome. African American infants also are more likely to have mothers affected by chronic stress both pre- and post-natally. Perhaps relatedly, African American offspring are disproportionately affected by neurodevelopmental delays. Taken together, these findings suggest that one important mechanism that may link prenatal and postnatal stress and African American infant brain development is the composition of the infant microbiome.

**Methods:**

In our ongoing longitudinal study, Maternal Stress and the Gut-Brain Axis in African American Infants (R01MD009746), we investigate associations between maternal prenatal and postnatal stress and the composition of the infant gut microbiome, in relation to cognitive and social-emotional development. We aim to recruit 300 African American mother-infant dyads, contingent on the mother’s previous participation in an associated prenatal cohort study: Biobehavioral Determinants of the Microbiome and Preterm Birth in Black Women (R01NR014800). Following enrollment, we assess infants at 1-week, and 3-, 6-, 12-and 18-months to collect: standardized assessments of infant neurocognitive and social-emotional development; questionnaire measures of infant feeding and health; observational data on maternal-infant interactions; maternal reports of postnatal stress; blood and saliva samples to evaluate maternal and infant psychoneuroimmunologic (PNI) function; and infant stool samples to characterize acquisition and trajectory of gut microbiome composition. Genetic variants of the major histocompatibility complex that may influence gut microbiome composition are also being evaluated.

**Discussion:**

This rich data set will allow future consideration of risk and protective factors that influence neurodevelopment in African American infants who are exposed to varying levels of prenatal and early life stress. Evidence for a mechanistic role of the microbiome would provide a framework for future clinical evaluations of preventative interventions (e.g.*,* probiotics, culturally-appropriate breastfeeding campaigns) that could potentially improve the health and development of African American children in infancy and across the lifespan.

## Background

While the brain’s regulation of gut function has long been recognized, only in the last decade has the bidirectional nature of this relationship been elucidated [1]. As a result of the Human Microbiome Project [2] evidence has mounted that gut-brain communication occurs via interactions between the gut microbes and established psychoneuroimmunologic (PNI) pathways, including: immunological (cytokines), endocrine (hypothalamic-pituitary-adrenal [HPA]), and neural (vagus) pathways [3, 4]. In addition, the gut microbiome may be an important link between our genes and exposure to psychosocial as well as environmental factors that influence susceptibility to neurodevelopmental and mental health outcomes [5]. Specifically of interest in our study, are the unique contributions of maternal prenatal chronic stress exposure and environmental toxicant exposure on infant outcomes.

### The microbiome-gut-brain Axis

Initial evidence suggests that prenatal stress exposure is associated with changes to the infant gut microbiome [6], as well as the composition of the adult gut microbiome in humans [7, 8]. Animal studies provide additional support for causal effects of prenatal stress [9]. For example, a decrease in the abundance of bacteria of the genus *Lactobacillus* in rat pup offspring develops after cortisone injections in pregnant rats [10]. In addition, in a study of rhesus macaques, experimentally manipulated stress in the prenatal period was associated with a lower abundance of bacteria of genera *Bifidobacterium* and *Lactobacillus* as well as increased rates of infection in the first six months of life [11].

### Fetal and infant gut microbiome: exogenous and endogenous influences

#### The first years

Although recent findings call into question the widely-accepted notion that the fetal gut microbiome is sterile [12], the preponderance of evidence continues to suggest that microbial colonization of the infant gut is largely established at birth, influenced by the composition of the mother’s vaginal microbiome for those infants delivered vaginally, and maternal skin or the hospital environment for those delivered surgically [13, 14]. Other notable contributors include infant and perhaps maternal genetic factors, and certain perinatal occurrences including antibiotic exposure [13]. The size and gestational age of the neonate also affects the composition of the gut microbiome [15]. Continuing over the next few months of life, infants begin to develop body site-specific microbiomes, with feeding patterns primarily influencing gut microbiome composition. The microbiome of breastfed infants is characterized by substantially higher abundance of *Bifidobacteria, believed to be beneficial to immune functioning* [16], whereas the formula-fed infant has a more diverse gut microbiota [17]. In addition to breast versus formula feeding, other household exposures including those provided to the infant by additional family members and pets are also likely to influence the early infant microbiome.

The next major influencers for the infant gut microbiome include the type and timing of the introduction of complementary foods in the first one-to-two years of life. During this phase of development the microbiome is a dynamic entity with each dietary juncture bringing new species and sometimes, eliminating others [18, 19]. As the infant enters the toddler stage, hospitalization and/or antibiotic exposures continue to influence the gut composition, with antibiotic exposure in particular frequently associated with substantial loss of diversity [20, 21]; depending on the infant and the timing of the exposure, the pre-established gut environment may remain altered long after the exposure.

#### Pre- and post-Natal stress exposure

Both the infant and adult microbiome may be influenced by maternal stress experienced during fetal life and after birth [6–9, 11]. Moreover, individual genetic factors are likely to moderate the influence of stress, including those related to the Human Leukocyte Antigen (HLA) genotype. In addition to their role in immune function, class II HLA genes are expressed throughout the developing fetal brain [22]. Class II HLA genotypes influence the immune response including the production of pro- and anti-inflammatory cytokines, with concern in particular related to the presence of certain HLA genotypes, which if present in preterm infants administered corticosteroids, are associated with immunosuppression and cytokine dysregulation [23, 24]. This period of reduced immune function may compromise microbiome development [25]. Further, prenatal stress may cause transient immunodepression during the time the microbiome is being established, again, with particular risk for infants with specific class HLA genotypes. The prenatal stress and microbiome association may also be affected by feeding, gestational size, mode of delivery, and antibiotics exposure as these factors (as detailed above) have substantial influences on the composition of the neonatal microbiome.

#### Fetal and infant microbiome and neurodevelopment

The gut microbiome has increasingly become a focus of clinical and preclinical studies of fetal and infant neurodevelopment. While effects in humans are still under investigation, rodent studies support that the gut microbiome modulates brain development, neurotransmitter systems, canonical signaling pathways, synaptic related proteins and behavior [26]. Rodent studies have noted deficits in social functioning [25] and working memory [27] in newborn microbiota-free animals. Notably, post-weaning microbial colonization of the gut in pups resulted in a reversal of these social deficits [25], suggesting that microbiome-associated developmental delays might be modifiable through treatment. Studies of germ free animals have noted mixed effects on anxiety, and some researchers have theorized that anxiety-related outcomes may be gene-dependent [28]. In humans, correlational studies have linked the neuro-developmental disorder autism to an abnormal gut microbiome composition [29], and a recent prospective study linked infant gut microbiome composition at one year of age to language scores at age two [30].

The first two years of life are a time of rapid changes in both the gut microbiome and the central nervous system (CNS). As described above, the ongoing communication between the gut, the brain, the HPA axis, and the immune system [12, 26] suggest that a myriad of postnatal factors influence the infant microbiome and its association with neurocognitive and social-emotional development. Preclinical studies provide evidence that these developmental systems impact one another and researchers are now theorizing potential far-reaching negative mental and physical health implications of early life disruptions in these systems [26].

To better understand these complex interactions, the proposed study examines the association between the infant gut microbiome and neurodevelopment, assessing factors that might moderate this association. For example, postnatal stress exposure is known to negatively affect brain development both through increased levels of cortisol as well as through disrupted immune functioning and the overproduction of cytokines, both pathways influenced by the microbiome in animal studies. In contrast, both breastfeeding and sensitive parenting might act as protective factors for infant neurodevelopment, influencing the gut microbiome and PNI processes in ways that promote healthy neurocognitive and social-emotional development [12].

#### Health disparity, neurodevelopment, and the microbiome

Health disparities begin in utero and continue after birth. Compared to white infants, African American infants are more than 1.5 times as likely to be born preterm (< 37 weeks), and more than twice as likely to experience infant mortality [31]. Increased chronic stress exposure during pregnancy and a differential susceptibility to prenatal stress are hypothesized to significantly contribute to these higher rates of preterm birth and infant mortality in African Americans ([32]; Institute of Medicine). Importantly, there is preliminary evidence that racial discrimination acts as a distinct form of stress in pregnancy and predicts adverse birth outcomes above and beyond other stressful life events [33]. As a result of a lifetime accumulation of chronic stress, African American women may also begin their pregnancies with a higher allostatic load, (accumulated physical impacts of stress on the body) translating into increased cardiovascular, metabolic, and neuroendocrine susceptibility to prenatal stressors [31].

Health disparities for African American infants continue postnatally and include higher rates of poverty and associated stressors, as well as lower rates of breastfeeding [34]. African American youth are also more likely than Caucasians to evidence neurocognitive delays [35]. This racial disparity persists or worsens across development, even when controlling for socioeconomic status [36, 37]. One recent study found that disparities in low birth weight and preterm birth explain a substantial proportion of the racial disparity in early developmental delays [37]. Parenting behaviors were also found to explain part of the association between African American race and attention problems at 24 months [38]. African American children also have higher rates of anxiety and aggression, suggesting increased postnatal social-emotional developmental problems as well [39]. Taken together these research findings suggest that the gut microbiome and the gut-brain axis may be critical contributors to the continued health disparities that negatively impact African American families.

Despite the burgeoning number of theoretical papers describing the microbiome and its potential role in infant neurodevelopment, there are only a handful of empirical studies that have tested the infant gut microbiome in association with prenatal stress and infant outcomes, and none that have examined these relationships in an African-American sample of infants. To address these knowledge gaps and assess the roles of stress, genetics, and the microbiome in neurodevelopment of African American infants, we have developed the Emory University African American Stress and Infant Gut Microbiome Cohort Study.

The objective of this report is to present the research design, data collection and laboratory methods of our the Emory University African American Maternal Stress and Infant Gut Microbiome Cohort Study, and to identify how this protocol will lead to the completion of the following Study Aims: to: 1) Characterize the relationship between maternal prenatal stress and infant gut microbiome at 1-week of age; 2) Evaluate the pathways between infant gut microbiome, PNI function, and infant neurocognitive and social-emotional development from 1-week to 18-months; and 3) Evaluate the associations among the infant gut microbiome, maternal caregiving and stress, and infant neurocognitive and social-emotional development from 1-week to 18-months.

## Methods

### Study design

The Emory University African American Maternal Stress and Infant Gut Microbiome Cohort Study enrolls women who have completed the Emory University Prenatal Microbiome Cohort study [40]. As described in detail previously, in this on-going prenatal longitudinal study, pregnant women are enrolled during their first trimester of pregnancy and followed through delivery, completing an assembly of stress and behavioral measures at enrollment and again during the third trimester of pregnancy. At each time point, maternal oral, vaginal, and gut microbiome samples are collected, as is blood for analysis of pro- and anti-inflammatory cytokines and genotyping. At the third trimester data collection, the women are asked if they are interested in continuing as part of a postnatal mother-infant dyad cohort: those who agree are consented at that time and contact is continued through email and text messaging, until the infant is born. Upon birth of their infant, mothers again provide informed consent for inclusion of their infant in the postnatal study. Mother-infant pairs subsequently are visited at home at 1-week, and 3-, 6-, 12- and 18-months post-birth. Currently, 175 women and infant dyads are enrolled; our aim is to recruit at least 300 women. The study was approved by the Emory University Internal Review Board (IRB) and the appropriate review councils for each hospital where prenatal recruitment occurs.

### Conceptual framework

**Fig. 1:**
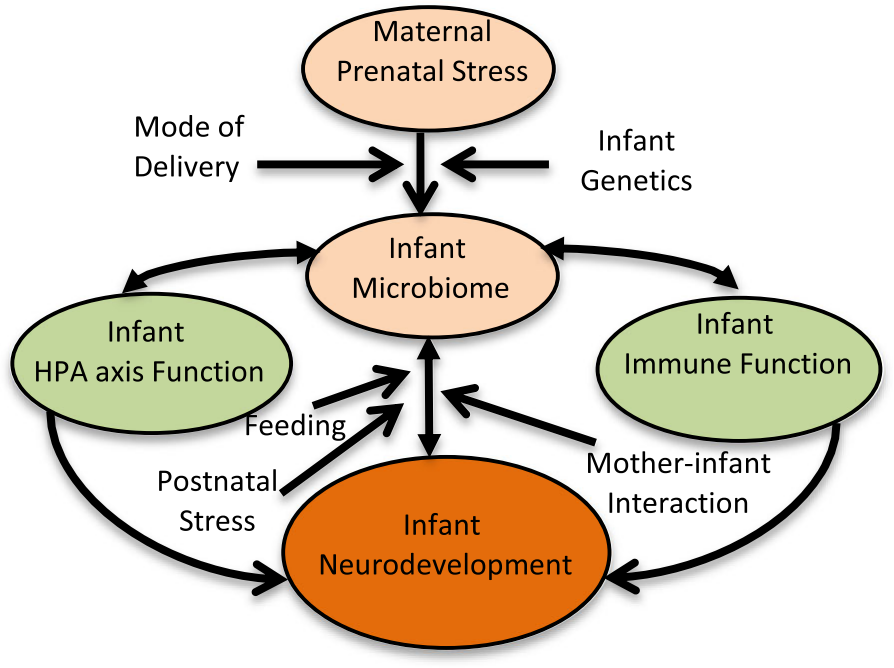
Conceptual Model

This study is guided by a conceptual framework that posits: maternal prenatal stress (operative within the socioeconomic context of African American women’s lives), impacts the infant microbiome, which in turn influences − and is influenced by – the infant HPA axis and immune system function. The infant microbiome, HPA axis and immune system together influence neurocognitive and socioemotional development (see Fig. 1). Mode of delivery, genetics, type of feeding, postnatal stress, and maternal-infant interaction are posited as moderators of this intergenerational risk process.

### Study population and sites

The proposed project leverages participant recruitment and perinatal data from an ongoing parent study (R01NR014800) [40]. Data collection for the infant cohort includes postnatal measures on mothers and infants through the first 18-months of life.

Pregnant African American women are recruited from prenatal care clinics of Emory Midtown and Grady Hospitals, which together provide prenatal care to ~ 3000 women annually. The parent study will recruit a final cohort of over 500 African American women. Characteristics of African American women delivering at these two hospitals are different. For Emory and Grady Hospitals, respectively, the percentage of African American deliveries to married women are 29 and 7%, to women with Medicaid are 68 and 91%, and with < high school education are 19 and 45%. Given that socioeconomic status is a determinant of the stress, nutrition, and health behaviors under study, the diversity across these two hospitals will provide us with sufficient variation in the biobehavioral factors to address the aims of the study.

Inclusion criteria for the parent study include: 1) Maternal African American race (via self-report); 2) Anticipated singleton pregnancy between 8 and 14 weeks’ gestation (verified by clinical record); 3) Ability to comprehend written and spoken English; 4) Age 18–40 years; and 5) No chronic medical conditions or use of chronic medications (verified by prenatal record). All women who participated in the parent study, whose pregnancy resulted in a live birth, whose infant is born without congenital disorders (e.g.*,* spina bifida, cerebral palsy), who have valid contact information, and who do not move out of the metropolitan Atlanta area are eligible for the infant follow up.

### Participant recruitment

Women who are completing prenatal visits associated with the parent study (at the second prenatal care visit occurring between 24 and 30 weeks) are provided information about the study by a member of the parent study staff. Participants who express interest in the infant follow-up study are encouraged to text the infant study coordinator at that time, and are provided the contact information. The infant study coordinator also are directly provided the list of names and contact information for all women who complete the second prenatal study visit and who consented to share this information. Continuing contact efforts consist of texts and phone calls, and women who express an interest in participating in the infant study are scheduled for enrollment approximately one week following their estimated date of delivery.

Women in the parent and infant studies move frequently and change phone numbers frequently. Efforts to reconnect include calling any listed contacts, and checks of White Pages. Ongoing efforts are made to enroll women in the study at any one of the study time points that they are contactable, unless or until participation is refused. Recruitment is ongoing and includes 185 study participants to date. Active refusals constitute 9%. Retention rates are approximately 80–85% throughout the infants’ first year.

### Data collection

Maternal prenatal stress data are collected two times during pregnancy (at the first prenatal care visit occurring between 8 and 14 and at a later prenatal care visit occurring between 24 and 30 weeks). Prenatal stress measures include self-reported stressful life events, perceived stress, perceived discrimination, state anxiety and depressive symptoms, as well as hair cortisol collection. Medical record abstraction is conducted by trained research staff for evidence of pregnancy infections, complications and treatments, and laboratory outcomes. Post-delivery medical record abstraction is detailed for birth outcome (gestational age, birth weight, size-for-age), delivery type, and complications.

Postnatal study visits occur at five times (1-week, and 3-, 6-, 12-and 18-months) post-birth and consist of biological samples, questionnaire data, and infant assessments. Data collection is conducted in participants’ homes or at the university laboratory by experienced research staff, trained in all aspects of the protocol. To the greatest degree possible, all home visits except the 1-week visit are scheduled for ±2 weeks of the designated time; the 1-week visit are scheduled for ±2 days. For infants born premature, the initial 1-week visit (and other follow up visits) are scheduled according to the corrected gestational age.

Enrollment and consent occur at the first postpartum study visit in which the dyad participates. Mothers provided consent for their own and their infant’s participation. Following consent, infant neurodevelopmental assessments (*NICU Network Neurobehavioral Scale* [NNNS] at 1-week [41] or Bayley Scales at 3-,6-,12-, and 18-month visits [42]) and growth measures (height, weight, and head circumference) are administered by trained research staff while the mother fills out questionnaires concerning her current demographic status, her home environment, her recent stress, her infant’s diet, and her and her infant’s sleep and health. At the 3-, 6-, 12- and 18- month visits, mothers and infants are then videotaped during a 5 min structured and a 5 min unstructured mother child interaction. At the one week and 18-month visits, blood is collected from the infant via heel stick and finger stick respectively, for the purpose of assessing cytokine levels (PNI functioning). Swabs are then used to collect infant cheek cells for DNA analyses at the 3-month visit.

At the end of each visit, the research team reviews the stool and saliva sample collection procedures, and provides the mother with the sample collection supplies for that visit. This ensures that samples are collected correctly. During a day selected by the mother for sample collection, texts are sent to the mother 5-min before each of the scheduled saliva collections (at wakening; 30 min after awakening and at 8:00 pm) to remind her to collect the saliva samples from herself and her infant, and the stool sample from the infant’s diaper. The research staff then schedule a time with the mother to pick up the samples. This sample retrieval visit also allows the research staff to collect any final assessments or questionnaires that were not collected during the main visit.

Data collection, including what samples are collected at each time over the course of the 18-month protocol, is presented in Table 1. All infant stool samples are obtained by the mother using four Catch All swabs. The mother uses each swab to collect a small amount of stool and then places the sample back into pre-labeled hard plastic case. The samples are stored in the home freezer until collection by the study team, ideally within 72 h. Research staff transfer the samples into labeled Mobio tubes at the home or transport them to the research laboratory where the specimens are transferred into labeled Mobio tubes and stored in a − 80 freezer. At the time of analyses, samples are defrosted, and the DNA extracted using the MoBio isolation Kit according to the manufacturer’s protocol. DNA yields are quantified with the ThemoFisher Broad Range Quant-It kit. After extraction, the V3-V4 region of the 16S rRNA gene is sequenced according to previously published and standard HiSeq protocols. To minimize batch effects, sequencing is run with longitudinal maternal and infant samples on the same HiSeq plate, and randomized by collection site and maternal pregnancy outcome, e.g. preterm birth yes/no).

**Table 1:** Biological and Questionnaire Data Collected at Each Time Point

Measure	Time Point	Group	Type of Assay/Analysis
Stool	1-week, 3-, 6-, 12-, & 18 month	Infant	Gut microbiome 16 rRNA gene sequencing
Blood	1-week & 18 month	Infant	Plasma cytokines
Saliva	3-, 6-, 12-, & 18 month	Infant, Mother	Diurnal cortisol, collected 3 times (at wake, 30 min after awakening, and 8 pm) & Salivary oxytocin
Buccal	1-week, 3, 6-, 12- month	Infant	HLA genotypes & DNA methylation
Questionnaires	1-week, 3-, 6-, 12-, & 18 month	Mother	Perceived stress, depression symptoms, anxiety
Infant Feeding Survey	1-week, 3-, 6-, 12-, & 18 month	Mother	Maternal report of infant feeding sources
Infant Food Diary	3,6 and 12 month	Mother	Record of infant food consumption in 24 h period
Infant + Maternal Sleep & health	3-, 6-, 12-, & 18 month	Mother	Maternal report of infant and mother’s sleep patterns and illnesses, diagnoses, medications
Parental Bonding	6 months	Mother	Maternal report of their mother’s parenting style for first 16 years of life.
Stressful life events & Neighborhood safety	12-month	Mother	Measures on new life stressors and neighborhood and home safety
Sociodemographic	3, 12-month	Mother	Questionnaire on sociodemographic variables
Videotaped Observation	3-, 6-, 12-, & 18 month	Infant, Mother	Maternal-infant interaction style
NNNS	1-week	Infant	Infant neurodevelopment
Infant Cry	1-week	Infant	Cry quality for neural integrity
Bayley Scales	3-, 6-, 12-, & 18 month	Infant	Infant cognitive and social-emotional development
Child Growth Measures	1-week, 3-, 6-, 12-, & 18 month	Infant	Infant growth trajectory
Maternal Cognitive Assessment	Once at any visit	Mother	Measures memory, visual processing and information retrieval

### Data management

Research assistants enter questionnaire and clinical data into pre-programmed fields that minimize data entry errors, via REDCap management software. For ready access and analyses, data are stored in separate but linked databases, which contain a unique ID for each dyad within each record so that data can be easily linked across databases.

### Statistical plan

Our initial data analyses will include: 1) determining the distributions of outcome measures and assessing whether data transformations are needed, 2) insuring that the underlying assumptions of statistical analyses are satisfied, 3) identifying potential co-linearity problems, 4) identifying potential outliers that require further investigation, 5) confirmatory psychometrics for established psychosocial and developmental measures. For categorical variables, we will check for sparse cells and regroup categories if necessary. In building multivariable models, we will check linearity assumptions for continuous predictors and consider higher-order terms as appropriate. For missing data, sensitivity analysis will be performed to evaluate the impact of missing data on results, with consideration for implementing multiple imputation of missing values. We will apply Bonferroni correction when appropriate to adjust for multiple comparisons. Analyses will be carried out using R (including OpenMX SEM, and lme4 mixed modeling packages) and SAS.

Methods to summarize the complexity of 16S rRNA data continue to evolve. Individual Operational Taxonomic Unit (OTU) abundance, richness, alpha (within sample) and beta (between sample) diversity, dysbiosis scores, phylogeny-based or phylogeny-independent measures, and principal components are all used in microbiome analyses. To characterize the trajectory of gut microbiome acquisition, we will first calculate the relative abundances of individual taxa (e.g. *Bifidobacterium or Lactobacillus)* or the ratio of abundance of two taxa (e.g. *Lactobacillus*, *Proteobacteria*), as well as estimation of the alpha diversity with a measure such as the Shannon Diversity Index. Subsequent exploratory analysis will use principal components analysis and Unifrac distances as alternatives means for characterizing gut microbiome. The Shannon Index ranges from 0 to ln(x) where x is the number of taxa measured, but can be standardized to range from 0 to 1; the abundance of individual taxa can be a proportion or captured as a count variable (with log number of reads as offset) with the possibility of excessive frequency of zero counts; finally the ratio of the abundance of two taxa may be skewed, but can be log-transformed to range from minus to positive infinity. Thus in all cases the gut microbiome for a given infant at each point in time can be measured using continuous or count-based statistics and analyses which will be adjusted accordingly to accommodate log transformation, Poisson or zero-inflated Poisson processes, and overdispersion.

To best capture the richness of the data, analyses considering maternal prenatal stress will be carried out in a structural equation modeling (SEM) framework reducing measurement error for the latent construct of maternal prenatal stress. Based on exploratory examination of the measures we may consider a single latent prenatal stress variable, or possibly two (e.g. chronic/life course stress and acute anxiety/perceived stress). We will then estimate the association between the latent variable(s) and outcomes. Postnatal maternal stress measures will also be combined in a similar manner to capture overall stress burden across infant developmental periods. PNI function will be evaluated as the absolute levels of each cytokine and as the ratios of pro- to anti-inflammatory factors (e.g.*,* IL1/IL10), and may be log-transformed if indicated. Diurnal cortisol from the three samples will be estimated as the area under the curve (AUC). The NNNS total score will be incorporated into our analysis framework as our primary measure of newborn neurobehavior function. The Bayley III standardized scores for the mental, motor and behavioral instruments will be considered independently to separately characterize neurocognitive and socio-emotional development. Repeated administrations of the Bayley-III also allow for the analysis of trajectories of growth over time from 3- to 18-months of age.

PLINK will be used to generate summary statistics for basic quality control determination of the array based genotypes. Only single nucleotide polymorphisms (SNPs) with a call rate > 99% and a Hardy-Weinberg equilibrium (HWE) p-value > = 10^− 5^ in controls will be included in the analysis. For each sample, we will also perform sex checks based on heterozygosity of X chromosome SNPs, evaluate autosomal heterozygosity, and eliminate SNPs that demonstrate non-random patterns of genotyping failure. Data from the 1000 Genomes Project indicate that multiple common class II HLA genotypes found in AAs are associated with birth outcomes, immune function and neurocognitive outcomes. These genotypes will be the focus of the primary analyses in the proposed project. HLA “genotypes” are derived from dozens of individual SNPs throughout each HLA gene. Consistent with standard approaches, we will impute non-canonical HLA genotypes (i.e. DQB1*0201) from the genome wide association study (GWAS) data and code each allele as 0 or 1 based on its presence or absence in each subject. The number of genotypes will be tested for association with each of the measures of microbiome variation. The remaining genome-wide data will be used to perform the quality control described above and to properly control for ancestry in the proposed analyses. We will test whether prenatal stress or any of our response or outcome variables are significantly associated with ancestry, and adjust as required if non-independence is revealed.

### Analysis for Aim 1

The association between maternal prenatal stress and infant gut microbiome will be tested using SEM with prenatal stress instruments as manifest or observed measures of latent stress, and taxa-specific abundance measures or alpha- or beta-diversity measures as the endogenous or outcome variable. Candidate causal confounders for this and subsequent aims will be considered using directed acyclic graph (DAG) theory and prior research. We will also consider modification of the effect of maternal prenatal stress on infant microbiome taxa abundance or diversity by one or more of several candidate variables: infant HLA genotype; caesarean versus vaginal delivery; gestational age at birth; breast versus formula feeding; and postnatal administration of antibiotics.

### Analysis for Aim 2

Preliminary testing will begin with a series of cross-sectional multivariable regression analyses testing the association between microbiome (e.g. Shannon Diversity Index) and PNI operationalized as the ratio of pro- to anti-inflammatory cytokines, or the cortisol AUC at each wave of follow-up. Next linear growth curve models with time invariant covariates and baseline microbiome predicting PNI trajectories will be fit to estimate the role of initial gut microbiome conditions on subsequent infant PNI trajectories. Finally fully longitudinal growth curve models including time-varying (e.g. microbiome measure trajectories) and time-invariant predictors will be estimated. Alternative mixed effects longitudinal models will be compared using Akaike Information Criterion (AIC) fit statistics. We will also test the association between PNI (now serving as a predictor variable) and infant development measured with the NNNS, and Bayley.

### Analysis for Aim 3

We will directly test the association between microbiome abundance or diversity, and trajectories in infant neurocognitive and socio-emotional development as measured using the Bayley. The modeling strategy will be similar to Aim 2 with initial exploratory and descriptive modeling followed by fitting a series of longitudinal growth curve models with time-varying and invariant covariates. Finally, we will include a series of candidate modifiers of the effect of microbiome on child development, operationalized as interaction terms. The candidate moderators include maternal post-natal stress, and maternal-infant interaction. These moderator models permit the estimation of possible heterogeneity of the association of microbiome and development.

### Power calculations

The study will enroll approximately 300 mother-infant dyads from the parent study for further follow-up. Because of the various complex relationships hypothesized in this project, power calculations were carried out using Monte Carlo simulations of hypothesized data generating processes permitting empirical estimation of coverage and power as a function of sample size and standardized effect sizes, *d:* $\frac{\left({\mu}_1-{\mu}_0\right)}{\sigma }$. Aim 1 concerns the association between prenatal stress (measured in the parent study) and infant microbiome at baseline for the follow-up study, thus the full sample will be available. Using multiple indicator variables for the latent construct of prenatal stress can reduce measurement error and improve power. For example we estimate 93% power to detect a correlation between prenatal stress and Shannon Index of diversity of *r = .22*, and 78% power to detect a correlation of *r = .18*. Aims 2 and 3 rely on analysis of repeated measures data at 1 week, 3-, 6-, 12-and 18-months; based on investigator experiences in longitudinal studies we anticipate at most 20% loss to follow-up resulting in N = 240 for longitudinal analyses. For example for Aim 3 we estimate 82% power to detect correlation in microbiome diversity and the rate of change (latent slope) in the Bayley of *r = 0.15*. Power is similar for other longitudinal hypotheses.

## Discussion

Our study is the first to our knowledge to examine the associations between prenatal maternal prenatal stress and the infant gut microbiome, maternal-child stress hormone levels, and neurodevelopment; it is also the first of its kind conducted in African American families. The use of standardized measures of stress and neurodevelopment will aid in the interpretation of our results concerning alternative operationalizations (e.g.*,* diversity indices, ratios, etc.) of microbiome composition. The sample gains strength by its inclusion of 100% African-American women as mothers. Our purpose it to characterize the hypothesized associations between stress, the microbiome and infant development *within* race, thus allowing us to follow the principles of health disparity science that suggest that to identify the underlying causes of a health disparity, research must first be conducted to identify pertinent risk and protective factors within the disparity group itself [43]. This allows researchers to identify effects related to socioeconomic status versus race/ethnicity. Thus, the completion of this study as designed is very likely to make an important contribution to the field by providing new information vital to understanding and ultimately eliminating the health disparity experienced by African American women and children.

## Data Availability

Not applicable.

## Abbreviations

AIC: Akaike Information Criterion
AUC: area under the curve
CNS: Central Nervous System
DAG: directed acyclic graph
GWAS: genome wide association study
HLA: Human Leukocyte Antigen
HPA: hypothalamic-pituitary-adrenal
HWE: Hardy-Weinberg equilibrium
IRB: Internal Review Board
NNNS: *NICU Network Neurobehavioral Scale*
OTU: Operational Taxonomic Unit
PNI: psychoneuroimmune
SEM: Structural Equation Modeling
SNPs: single nucleotide polymorphisms
